# Dr. Walker, on the Report of the Fulwood's Rents Case

**Published:** 1805-01-01

**Authors:** John Walker

**Affiliations:** Salisbury Square


### *To the Editors of the Medical and Physical Journal.*

GENTLEMEN,

HAVING in some of the preceding Numbers of your Journal, made observations upon the case in Fulwood's Rents, I take the liberty to offer a few remarks upon that report of the committee, which was noticed by you in your last number. In doing this, I hope that every gentleman who composed that committee, (to a great part of which I owe my present situation) and the respectable characters of whom any mention may be made in my examination of the report, will believe me to be actuated by the sole motive of endeavouring to rescue the character of *genuine* vacciola from the suspicion (which I conceive to be ill founded) of not possessing sufficient efficacy to preserve the constitution from the future infection of the small-pox. The report of this committee must be considered as an important publication, which will be sought after with great eagerness in different parts of the world. Those celebrated characters, De Carro, Odier, Sacco, and other distinguished men, who have been the instruments of propagating the happy discovery through extensive tracks of the globe, will most certainly read with surprize and great concern, the concluding opinion of the committee, viz. " That there seems no reason to question the regular progress of the vaccination in Nancy and Mary Hodges; nor the existence of the small-pox more than two years afterwards in the latter." The great importance which must be attached to this statement, will most certainly excuse any observations which I may make upon any part of the Report; and I assure myself, that the authors will be exceedingly happy if it can be made to appear highly probable, that either the former or the latter part of their statement is ill founded.

Were I to take up this subject as a casuist, I might object to the manner in which the experiments of the inoculations from Mary Hodges were conducted. I might make some inquiries with respect to the instrument used, and the close inspection of the committee at the time of the matter being taken from Mary Hodges, and inserted into H. A. a boy aged eight months, Case 3; and Mary Robins, aged five months, Case 4. The other inoculations being of the second generation, can only be considered as dependant upon the validity of the two first cases. I migh ask hen, in the first place, what lancet did Friend Morgan use? Was it one furnished to him by the committee; a new one, purchased on purpose to avoid any chance of other matter being attached to it?

Friend Morgan, it appears from the report, had been attending patients with the small-pox, perhaps inoculating others for that disease, (Vide Report, page 10): Did he use any lancet which had ever before been soiled with variolous matter? *

The presence of any part of the committee during the second series of inoculation, is not of any consequence; all the importance of the report attaches to the first series, and rests entirely upon the faithfulness and accuracy of the inoculators, who alone took, and kept, and applied the matter. These are circumstances which must be remarked by every stranger. I know Morgan too well, and have too high a respect for his ingenuousness of character, to suspect him to be capable of exercising any legerdemain in his experiments; but this gentleman is not known to all the various characters who will investigate the report, and therefore, arguing as a perfect stranger, or casuist, I might say, there is a defect in the record, for it is only in the mouth of two or three witnesses that every word should be established.

Notwithstanding the want of vigilance, which appears to me to have marked the proceedings of the committee on this most important affair, there is certainly a very great shew of it, in exhibiting copies of the register of the case, when under inoculation for the cow-pock; yet, what end can it answer to exhibit the cases from which the original inoculation was made? If the committee had exhibited from the register, cases of perfect vacciola by inoculation (with clean lancet) from Mary Hodges, they would have taken the only perfectly tenable *extra se* ground on which to erect their conclusion, that in Mary Hodges the "progress of vaccination had been regular."

---

* I remember it once happened to me, that I had ten or twelve subjects to inoculate, when the sun was set, and candles not yet lighted; that I had a perfect case to inoculate from, but I thought the pock was too far advanced; I had no other, and ventured to use it. Inoculating each arm, I made more than twenty insertions of matter. I saw the subjects in a week, and one pock only was produced; the marks of the other punctures were all passed away. I found it had been the third insertion of my lancet which had produced the perfect effect; and concluded that the instrument, in constant use, had had a particle of active matter, dry or hard, on its point; that, on the first and second insertion, it was not yet dissolved; that, on the third, it had been sufficiently softened to be wiped off from the lancet and left behind in the puncture.

Their

Their proof, they say, rests on the following evidences: 1st, The register. 2dly, The declaration of the inoculator, who considers the appearances and progress of vaccination to have been perfectly regular and satisfactory. 3dly, The cicatrices or marks remaining on their arms.

The first of these evidences, viz. the register, seems by far the strongest offered in proof of the vacciolation having been complete. The recollection of any officer at a public institution extending to individual cases, (unless there have been something peculiar in them) would afford an instance of capaciousness, and of retention of memory, beyond the ordinary stretch of the human faculty. Cicatrices, not distinguishable from those which are left by inoculation, are produced from other causes, from a wound by a rusty nail, or from any other destruction of integument.

But, respecting the register itself, I see in it marks of vacciola not having yet been perfectly understood; for, at an early period, I find remarked, as a peculiarity, "*no pustules*," though no pustules is what we regularly expect, and what we find in vacciolation. I have seen "pustules," it is true, and had much anxiety about them; but it was where I had absolutely to refuse the gold which the confiding Spaniards pressed upon me, even when I told them my suspicions. (The opinions in my letter to the Governor Fox, page 54 of your last Number). These pustules, or spurious pocks, were elevated, contained lymph, and had a blossom, but the blossom was not the areola of the cow-pock; it was undefined and indistinct. It is to be feared, that thousands of such degenerated pocks may have been produced in early practice, before the beautiful vacciola was so generally known as it now happily is.

Has Mary Hodges undergone some such partial effect from vacciolation as the young Spaniards, who afterwards suffered so little from the small-pox? If so, she may also have had so imperfect a variola as to cause many of us to think that it was only varicella which was present.

Let not my worthy friend, the respectable gentleman who inoculated the child, conceive that any thing like disparagement is meant in these observations. So late as the year 1801, I had great pleasure in inoculating from a subject so far advanced, that he complained of pain in his arm-pits. Even then, I erroneously thought it a proof of its being a perfect case, though those who have previously had the small-pox, are sometimes known to endure much in this way from the inoculation of vacciola. I inoculated

 from from him with peculiar pleasure, but had the mortification to produce from him, in that late stage, only pustules or spurious pocks.

If the conjectures of the committee be right, that Ann and Mary Hodges have been perfectly vacciolated; that Mary has since had small-pox, and that Ann also has had it, according to the description of the mother, and the conclusion of the medical practitioner who attended her; If, I say, the two sisters inoculated, one in 1800, the other in 1802, have, now, contrary to the general experience, been found susceptible of any effect from variolous contagion, there must be in their family, as has been suggested to me by an experienced practitioner, some strange peculiarity of constitution, some singular susceptibility of infection. On this, the committee have offered no ideas, have not entered into any inquiries respecting what may have been the peculiar experience of the family of the father and mother under small-pox; whether they may have been affected by it oftener than once.

To conclude, if, in a few rare instances, persons, having undergone the natural or the inoculated small-pox, have afterwards taken the disease by infection, we may very naturally expect a susceptibility of the variolous infection in a few rare instances, after the cow-pock. This susceptibility, however, for any thing the committee have yet published, "remains to be proven."

*Salisbury Square*, 8, *xii*, 1804. JOHN WALKER.

P. S. On finishing the above, arrived the letters of the day. "Notwithstanding," says a correspondent north of the Tees, "Report of vaccine influence having failed in a late instance, you will have the goodness to forward me a fresh supply of matter; being assured, from the first introduction of the practice, that the fault depends entirely upon the inoculator's inattention or want of information."

"I will thank you for any new information, as the report gains credit here, and consequently arrests the attention of the parents who have inoculated with vaccine fluid. I am, &c."

The above may prove the fitness of your making haste to shew, if it can be shewn, that the committee may have possibly made some conclusions which are incorrect, on a subject where so very little of reasoning can be employed. The "*morbi, semel tantum in decursu vitæ aliquem afficientes,*" must ever baffle every attempt to explain or even to offer any comprehensible theory on this peculiarity. Their history can never be composed but of simple chapters of facts.

